# Integrated Bioinformatics Analysis of Potential mRNA and miRNA Regulatory Networks in Mice With Ischemic Stroke Treated by Electroacupuncture

**DOI:** 10.3389/fneur.2021.719354

**Published:** 2021-09-10

**Authors:** Chunxiao Wu, Lijun Zhao, Xinrong Li, Yingshan Xu, Hongji Guo, Zifeng Huang, Qizhang Wang, Helu Liu, Dongfeng Chen, Meiling Zhu

**Affiliations:** ^1^Shenzhen Hospital of Integrated Traditional Chinese and Western Medicine, Guangzhou University of Chinese Medicine, Guangdong, China; ^2^The Research Center of Basic Integrative Medicine, Guangzhou University of Chinese Medicine, Guangzhou, China; ^3^Clinical Medical of Acupuncture, Moxibustion and Rehabilitation, Guangzhou University of Chinese Medicine, Guangzhou, China

**Keywords:** electroacupuncture, ischemic stroke, miRNA-mRNA networks, bioinformatics analysis, molecular mechanisms

## Abstract

**Background:** The complicated molecular mechanisms underlying the therapeutic effect of electroacupuncture (EA) on ischemic stroke are still unclear. Recently, more evidence has revealed the essential role of the microRNA (miRNA)–mRNA networks in ischemic stroke. However, a systematic analysis of novel key genes, miRNAs, and miRNA–mRNA networks regulated by EA in ischemic stroke is still absent.

**Methods:** We established a middle cerebral artery occlusion (MCAO) mouse model and performed EA therapy on ischemic stroke mice. Behavior tests and measurement of infarction area were applied to measure the effect of EA treatment. Then, we performed RNA sequencing to analyze differentially expressed genes (DEGs) and functional enrichment between the EA and control groups. In addition, a protein–protein interaction (PPI) network was built, and hub genes were screened by Cytoscape. Upstream miRNAs were predicted by miRTarBase. Then hub genes and predicted miRNAs were verified as key biomarkers by RT-qPCR. Finally, miRNA–mRNA networks were constructed to explore the potential mechanisms of EA in ischemic stroke.

**Results:** Our analysis revealed that EA treatment could significantly alleviate neurological deficits in the affected limbs and reduce infarct area of the MCAO model mice. A total of 174 significant DEGs, including 53 upregulated genes and 121 downregulated genes, were identified between the EA and control groups. Functional enrichment analysis showed that these DEGs were associated with the FOXO signaling pathway, NF-kappa B signaling pathway, T-cell receptor signaling pathway, and other vital pathways. The top 10 genes with the highest degree scores were identified as hub genes based on the degree method, but only seven genes were verified as key genes according to RT-qPCR. Twelve upstream miRNAs were predicted to target the seven key genes. However, only four miRNAs were significantly upregulated and indicated favorable effects of EA treatment. Finally, comprehensive analysis of the results identified the miR-425-5p-Cdk1, mmu-miR-1186b-Prc1, mmu-miR-434-3p-Prc1, and mmu-miR-453-Prc1 miRNA–mRNA networks as key networks that are regulated by EA and linked to ischemic stroke. These networks might mainly take place in neuronal cells regulated by EA in ischemic stroke.

**Conclusion:** In summary, our study identified key DEGs, miRNAs, and miRNA–mRNA regulatory networks that may help to facilitate the understanding of the molecular mechanism underlying the effect of EA treatment on ischemic stroke.

## Introduction

Ischemic stroke is characterized by the loss of blood and oxygen in an area of the brain, resulting in damage or death of brain neural cells. Ischemic stroke is also one of the leading causes of long-term disability and death worldwide (1). The current therapies for ischemic stroke include thrombolysis, endovascular treatment, anticoagulation drugs, surgery, and rehabilitation (2, 3). However, most patients miss the optimal time window for best treatment results and develop long-term motor and sense disabilities, which make it difficult to solve these problems (4, 5). Therefore, it is vital to find an alternative and effective method for treating ischemic stroke.

Electroacupuncture (EA) is an alternative therapy that combines acupuncture and modern electrotherapy, and it is beneficial for promoting sense and motor function recovery in ischemic stroke (6, 7). Previous studies demonstrated that EA could significantly reduce neurological deficit scores, decrease the percentage of brain infarction area, and protect brain cells from further damage in ischemic stroke patients or animals (6, 8–10), These findings suggest that EA is an effective method for treating ischemic stroke, especially for improving neurological functions and quality of life (11, 12). The potential mechanisms of EA in ischemic stroke might relate to multiple biological processes, including neurogeneration, inflammatory response reduction, and signaling pathway regulation (7, 13, 14). However, the molecular mechanisms of EA in ischemic stroke, especially the regulation of key genes and miRNA networks, are still largely unknown.

To further explore the potential complicated molecular mechanisms in a variety of diseases (such as stroke, cancer, and Parkinson's disease), high-throughput RNA sequencing (RNA-seq) was used to identify key genes and functional pathways, and construct RNA-mediated networks (15–17). Previous studies suggested that microRNAs could negatively regulate target genes and be involved in biological processes (cell apoptosis, proliferation, and inflammation) of ischemic stroke (18–20). In addition, previous studies also revealed that mRNAs, microRNAs, lncRNAs, or circRNAs composed of different RNA-mediated networks regulated vital cellular mechanisms associated with ischemic stroke (21, 22). EA exerts a protective effect in ischemic stroke by regulating related pathways or key miRNAs, according to recent studies (23, 24). However, a systematic analysis of novel key genes, microRNAs, and miRNA–mRNA networks regulated by EA in ischemic stroke is still lacking.

Therefore, we generated a middle cerebral artery occlusion (MCAO) mouse model and performed RNA sequencing to analyze mRNA profiles in the brain tissue of mice subjected to EA and mice without EA treatment. Differentially expressed genes (DEGs) and key microRNAs were investigated in the EA and control groups. Functional annotation and protein–protein interaction (PPI) analyses were performed to explore potential mechanisms in ischemic stroke. Hub genes and predicted miRNAs were selected to verify RNA expression by RT-qPCR. Finally, miRNA–mRNA networks were constructed (established by upstream microRNAs and targeted key genes) to regulate ischemic stroke by EA therapy after a comprehensive analysis.

## Materials and Methods

All animal procedures followed the National Institute of Health guidelines and were approved by the Animal Welfare and Ethics Committee of Guangzhou University of Chinese Medicine.

### Animals

Adult male C57B6J/L mice (8–10 weeks old, 20–25 g, SPF grade) were obtained from the experimental animal center of Guangzhou University of Chinese Medicine. The mice were housed in a temperature, humidity-controlled condition with 12-h light–dark cycles and allowed free access to food and water. The mice were randomly assigned to each group using a random number generator in SPSS 25.

### Middle Cerebral Artery Occlusion Model Establishment

Treatment allocation was blinded when transient occlusion of the middle cerebral artery (tMCAO) was induced. All surgical procedures were conducted under isoflurane anesthesia to minimize injury. The mice were subjected to transient focal cerebral ischemia according to previously described methods (25). A nylon thread with a silicon-coated tip, 0.20 mm in diameter, was inserted into the middle cerebral artery (MCA) and anterior cerebral artery to block the blood. Following 60 min of occlusion, the nylon thread was retracted to allow reperfusion, while in the sham-operated group, an identical surgery was performed but without the nylon suture inserted. After surgery, anti-inflammatory drugs (carprofen 10 mg/kg) were injected to alleviate post-operative discomfort, and the post-operative mice were placed on a heated pad to prevent hypothermia and death. Finally, the mice were transferred to a cage for single housing and allowed free access to water and food. The mice that were excluded out of the study were those that showed sub-arachnoid blood at the time of sacrifice or died early before the endpoint of treatment duration, and the animals were sacrificed 7 days post-injury.

### Electroacupuncture Intervention

The mice were treated with EA after the MCAO model was established. DU20 (Baihui), DU14 (Dazhui), ST36 (Zusanli), and LI11 (Quchi) in the affected limbs were selected as EA acupoints for treating ischemic stroke. Mouse acupoints were located according to the Acupoint Standard for Experimental Animals as previously described (26). The location of the selected acupoints were listed as follows: DU20 (Baihui), at the middle point of the parietal bone of mice; DU14 (Dazhui), the posterior midline in the depression below the spinous process of the seventh cervical vertebra; LI11(Quchi), located at the depression medial to the extensor carpi radialis, at the lateral end of the cubital crease; ST36 (Zusanli), located at the proximal one-fifth site of the craniolateral surface of the leg, distal to the head of the tibia, in a depression between the muscles of the cranial tibia and the long digital extensor. Acupuncture needles (with 0.16^*^7 mm) were inserted at a depth of 2 mm. Acupuncture needles were connected with a HANS nerve stimulator (HANS-200E, Jisheng Medical Technology, Co., Ltd., Nanjing, China). The stimulation intensity of HANS-200E was 1 mA, the stimulation frequency was 2 Hz, and the duration of EA treatment was 20 min once daily for seven consecutive days.

### Behavioral Test

The mice were pretested with behavioral tests the day before MCAO surgery, and then these were performed again at 7 days post-surgery. All behavioral testing and scoring were carried out by experimenters who were blind to the treatment group. The detailed methods of the behavioral tests are shown below.

#### Adhesive Sticker Removal Test

The protocol of this behavioral test was based on methods previously described (27). In brief, the mice performed a 5-day training before the formal test, and then they were placed in the chamber, and an adhesive sticker was applied to the affected forelimbs. Two scores including sense score and the motor score were recorded during the test. First, we recorded the time taken for the mice to notice the stickers on their forepaws (represented by the mouse shanking its paw and bringing it to its mouth). Then we calculated the time taken for the mice to remove the adhesive stickers from their wrists. The mice were limited to a maximum of 300 s to remove the adhesive sticker. Behavioral tests of all groups were conducted before MCAO surgery and after 7 days of EA treatment.

#### Grid Walking Test

Grid walking test, also known as foot-fault test, was performed according to the previously described methods (28, 29). Briefly, the mice were placed on an elevated, leveled grid with openings (with 15 mm ^*^ 15 mm grid squares) and allowed to move freely across the grid for 5 min. First, a foot fault was recorded when the paw falls from or slips off the rung, and the total number of steps it took to cross the grid was also quantified. Then the foot-fault index of the affected forelimb in each group was calculated as foot-faults/total steps ^*^ 100%. The mice were placed on the grid prior to the test for habituation and then tested before and at 7 days after MCAO surgery.

### Measurement of Infarct Area

The mice in each group were sacrificed 7 days post-surgery. The mice under deep anesthesia were transcardially perfused with ice-cold phosphate-buffered saline (PBS) and followed by 4% paraformaldehyde. Then the brains were quickly removed and fixed in 4% formaldehyde solution at 4°C overnight and dehydrated in 15%−30% sucrose until they sank. The samples were cut into 40-μm sections by a cryostat vibratome. For each brain, every sixth section (anterior and posterior of bregma), covering mainly the striatum, was selected for immunofluorescence to measure the infarct volume. In brief, the sections were incubated with primary antibody of rabbit anti-NeuN (Rabbit polyclonal, 26975-1-AP; Proteintech, USA; 1:500) overnight at 4°C. Then the sections were incubated with appropriated secondary antibody of goat anti-rabbit conjugated to Alexa Flour 488 (Abcam, USA; 1:1,000) for 2 h at room temperature. The images were acquired by a Nikon confocal microscope. The brain loss volume was represented by the loss of neuron. Therefore, the percentage of cerebral infarction volume in mice brain was calculated as the following equation: infarction volume % = (sum of infarction size of each slice)/(sum of area of each slice of brain) × 100%, and the loss area of neuronal cells were quantified by the Fiji ImageJ software.

### RNA Extraction and RNA-Sequencing

Briefly, brain cortex tissues were obtained from each group (*n* = 3/group) at 7 days timepoint, total RNA was extracted from the brain tissues using TRIzol (Invitrogen, Carlsbad, CA, USA) following the protocol of the manufacturer. Subsequently, RNA was quantified and qualified using a NanoDrop and Agilent 2100 bioanalyzer (Thermo Fisher Scientific, MA, USA). mRNA was purified by using oligo(dT)-attached magnetic beads, and then RNA-sequencing libraries were constructed according to the instructions of the manufacturer. The final library was amplified to make DNA nanoballs (DNBs) of more than 300 bp in length. DNBs were loaded into the patterned nanoarray, and single-end 50-base reads were generated on the BGISeq500 platform (BGI-Shenzhen, China).

### Differentially Expressed Gene Analysis

The sequencing data (raw data) were filtered with SOAPnuke (v1.5.2), and quality control (QC) was performed using FASTQ (30). Afterward, HISAT2 (v2.0.4) was applied to map the clean reads to the reference genome (31). The clean reads were aligned to the reference coding gene set using Bowtie2 (v2.2.5) (32), and then RSEM (v1.2.8) was used to calculate the expression level of genes (33). Finally, DEG analysis was conducted using DESeq2 (v1.4.5) with a Q value smaller than 0.05 (34).

### Functional Enrichment Analysis of Differentially Expressed Genes

Gene Ontology (GO) and Kyoto Encyclopedia of Genes and Genomes (KEGG) pathway analyses were enriched using the phyper function of the R package to better reveal the functions and pathways of DEGs regulated by EA in ischemic stroke.

### Protein–Protein Interaction Network Construction and Hub Gene Identification

To obtain the interactions of the target genes and miRNAs, a PPI network of DEGs was predicted and constructed by using the STRING online database. The combined scores larger than 0.4 were considered a significant interaction. Cytoscape (version 3.7.2) was applied to visualize the network and distinguish the hub genes. Hub genes are considered to have crucial roles in the network and were investigated by the degree calculation of cytoHubba in Cytoscape. The top 10 ranking degree scores were selected as hub genes.

### Prediction and Construction of an miRNA–mRNA Network

Upstream miRNAs of hub genes were predicted by employing the miRTarBase database. Herein, we identified 10 hub genes and predicted microRNA interactions based on microarray, Western blot, reporter assay, qPCR, and NGS validation methods in the miRTarBase database. Then, the miRNA–mRNA information was extracted, and visualization of the miRNA–mRNA networks was constructed using Cytoscape software.

### RT-qPCR Validation Analysis

Total RNA was extracted from brain tissue samples using an EastepTM Super Total RNA Extraction Kit (Cat. No. LS1040, Promega Corporation). The ratio of absorbance at 260 and 280 nm is used to assess RNA quality (NanoDrop spectrophotometer, CA, USA). Related qPCR primers of the target genes and miRNAs were designed, and the specificity was checked by using Primer-Blast. Then total RNA was reverse transcribed into cDNA by a reverse transcription kit [PrimeScript RT reagent kit (Cat. No. RR600A, Takara Bio, Inc.) or miDETECT A TrackTM miRNA qRT-PCR Starter Kit (Guangzhou RiboBio Co., Ltd.)]. RT-qPCR was accomplished according to the instructions of the kit and performed on a Bio-Rad C1000 Touch™ Thermal Cycler. Relative gene and miRNA expression was quantified based on the 2^−ΔΔCT^ method. β-actin and U6 genes served as housekeeping genes for mRNA and miRNA, respectively. Brain tissues were obtained from each group (*n* = 3/group), and each experiment was conducted in triplicate. The primers are presented in the Supplementary Table 1.

### Fluorescent *in situ* Hybridization and Immunofluorescence

The localization of miRNAs in the brains of mice in each group were assessed by FISH and IF. In brief, the brains of the mice were quickly removed and fixed in 4% formaldehyde solution at 4°C overnight and dehydrated in 15–30% sucrose until they sank. Coronal sections were stained for FISH and IF analysis. The frozen sections were dried and fixed in 4% paraformaldehyde for 10 min, then digested in proteinase K (20 μg/ml) for 20 min. Hybridization buffer was added and incubated 1 h for prehybridization, then the prehybridization solution was removed, the targeted miRNA probe hybridization solution (500 nM) was added, and the sections were incubated overnight at 42°. After washing, the sections were blocked with blocking serum for 30 min. For immunostaining of FISH tissue, sections were incubated with the first antibody [NeuN (Rabbit polyclonal, 26975-1-AP; Proteintech, USA; 1:500), GFAP (Rat Monoclonal Antibody, Thermo Fisher Scientific, 13-0300, 1:1,000)], and then followed by incubation in a fluorescent with the second antibody. Finally, the sections were stained with DAPI for 8 min to visualize the nuclei. Slides were imaged on Nikon confocal microscope and analyzed using the ImageJ software.

### Statistical Analysis

Behavioral tests, measurement of infarct area, and RT-qPCR data are presented as the means ± SEM and were analyzed with one-way ANOVA and Bonferroni (if equal variances assumed) or Dunnett's T3 (if equal variances not assumed) *post-hoc* test in SPSS25. FISH and IF data were analyzed with unpaired *t*-test in SPSS25. A *p* < 0.05 was considered statistically significant.

## Results

**Figure 1:**
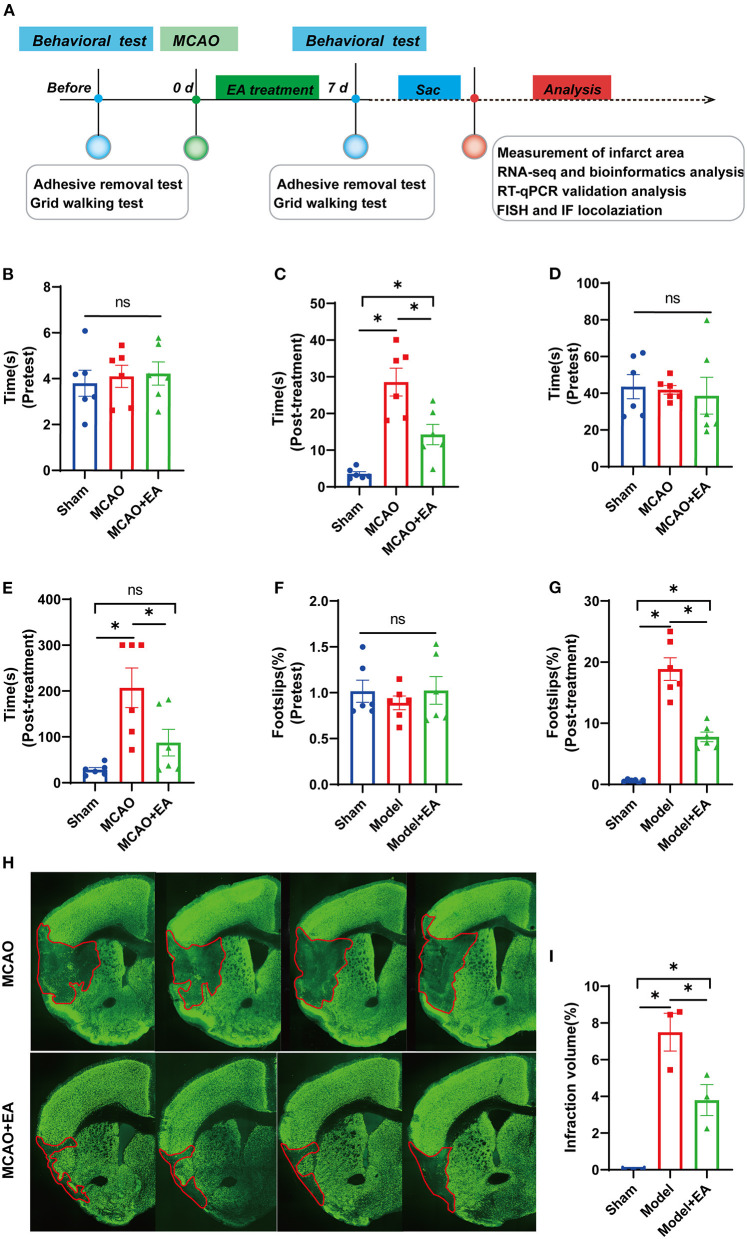
Electroacupuncture (EA) alleviates neurological deficits of middle cerebral artery occlusion (MCAO) mice. **(A)** A timeline schedule of the experimental protocol. **(B)** The time taken for the mice to notice the stickers on their forepaws among the three groups before MCAO surgery (*n* = 6 mice/group). **(C)** The time taken for the mice to notice the stickers on their forepaws among the three groups after 7 days of EA treatment (*n* = 6 mice/group). **(D)** The time taken to remove adhesive stickers among the three groups before MCAO surgery (*n* = 6 mice/group). **(E)** The time taken to remove adhesive stickers in the three groups after 7 days of EA treatment (*n* = 6 mice/group). **(F)** The percentage of foot-faults of affected forelimb in each group before MCAO surgery (*n* = 6 mice/group). **(G)** The percentage of foot-faults of affected forelimb in each group at 7 days post-surgery (*n* = 6 mice/group). **(H)** Representative NeuN stained sections from MCAO + EA and MCAO control group following 7 days of ischemic stroke. **(I)** The percentage of cerebral infarction volume of mice brain in each group (*n* = 3 mice/group). Data are represented as means ± SEM. One-way ANOVA followed by Bonferroni *post-hoc* test was used for statistical analysis. *p < 0.05, ^ns^*p* > 0.05.

Two mice that underwent MCAO died within 36 h to 7 days of surgery, and one mouse in the sham group immediately died because of the surgery accident. No other mortality was found in the sham group within 7 days post-operation. Finally, a total of 27 mice were included for further evaluation (nine mice in each group). A timeline diagram illustrating our study is shown in Figure 1A.

### Electroacupuncture Improves the Sensorimotor and Motor Coordination Function of Middle Cerebral Artery Occlusion Mice

Before MCAO surgery, there was no significant difference in the time taken to sense and remove adhesive stickers among the three groups, which indicates better comparability (all *p* > 0.05, Figures 1B,D). After MCAO and EA treatment, the neurological function (the less time to sense and remove stickers, the better motor function) in the EA and sham groups was significantly better than that in the MCAO control group. This suggests that EA could reduce neurologic deficits and improve sensorimotor functions after ischemic stroke (Figures 1C,E).

The foot-fault test was mainly used to detect both sensorimotor function and motor coordination of limb functioning in ischemic stroke. As shown in Figure 1F, no significant difference was observed in the percentage of footslips made by the mice among the three groups before MCAO surgery, which indicates better comparability (*p* > 0.05). However, all mice in the MCAO groups showed significantly more forelimb slips than those in the sham group at 7 days post-lesion. Furthermore, MCAO + EA mice had a lower percentage of footslips than the MCAO control group (*p* < 0.05, Figure 1G).

### Electroacupuncture Decreases the Infarct Volume of Middle Cerebral Artery Occlusion Mice

As shown in Figure 1H, compared with the sham group, the MCAO groups all had larger infarct volumes at 7 days after MCAO surgery (all *p* < 0.05), whereas the MCAO + EA group significantly reduced the percentage of brain infarction area compared with MCAO control group (*p* < 0.05, Figures 1H,I).

### Gene Expression Analysis

After EA intervention, we extracted total RNA from the MCAO control group and MCAO + EA group. RNA sequencing of each sample was assessed by using the DNBSEQ platform. Sample reads ranged from 44.25 to 44.71 million, and the total mapping ranged from 95.73 to 96.09%. Then, we calculated gene expression represented by fragments per kilobase of transcript sequence per million base pairs sequenced (FPKM) of each sample. The boxplot figure demonstrated that the distribution of FPKM expression was roughly consistent for each sample, which indicates that the RNA-seq data were reproducible and reliable (see Supplementary Figure 1).

### Screening for Significant Differentially Expressed Genes

**Figure 2:**
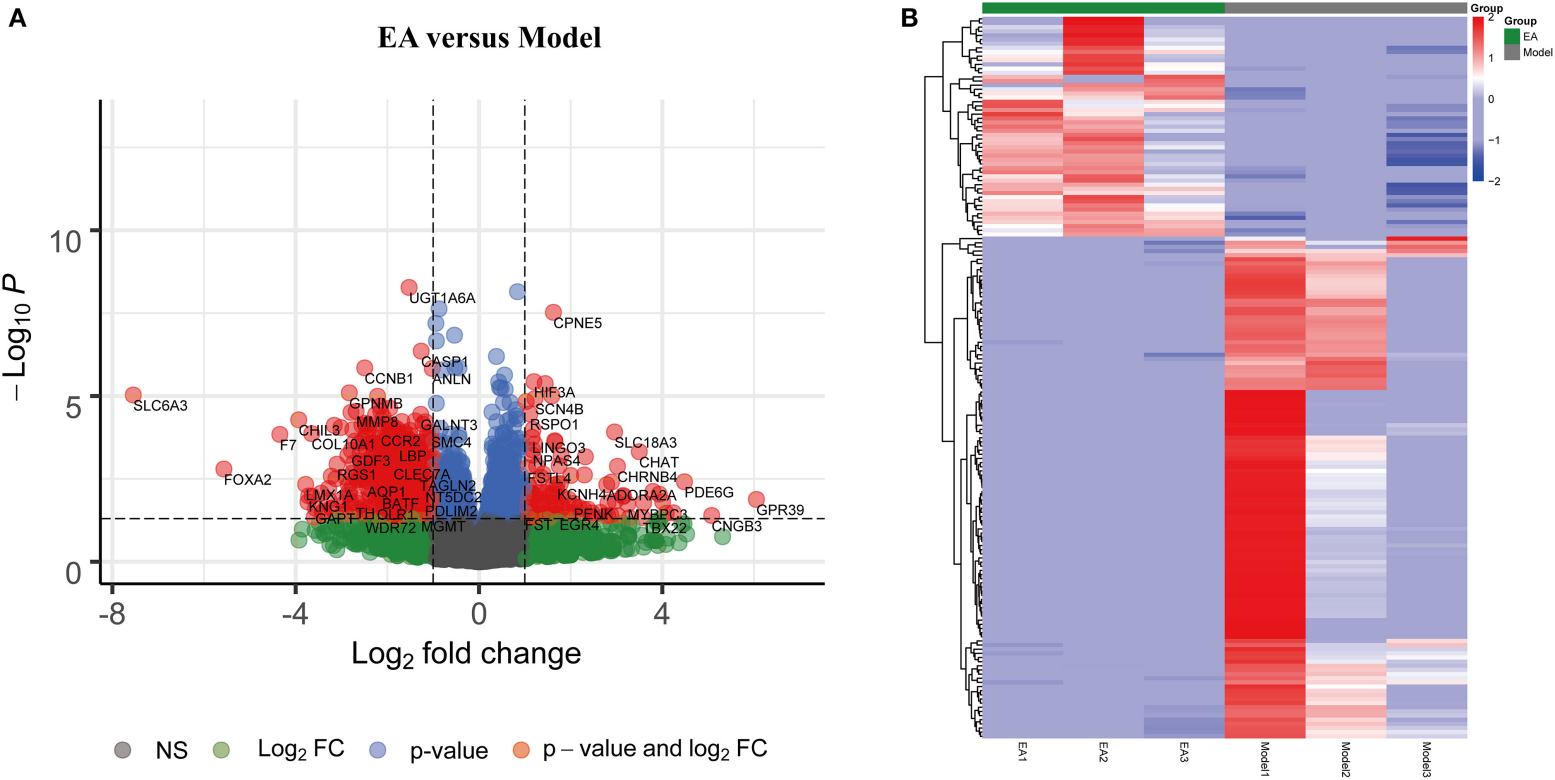
Significant differentially expressed genes (DEGs) between the EA and MCAO control groups. **(A)** Volcano plot showing significant gene expression profiles in the MCAO + EA group compared with the MCAO control group. Red points and blue points represent downregulated and upregulated DEGs, respectively. **(B)** Heat map and hierarchical clustering of DEGs between the MCAO and MCAO + EA groups. The deeper red color shows the higher gene expression, while the deeper blue color indicates the lower expression. *n* = 3 mice/group.

To further identify significant DEGs between the MCAO control and EA groups, the DESeq2 R package was used to analyze differentially expressed genes. |log_2_FoldChange| > 0 and adjusted *p* < 0.05 were regarded as criteria to search for significant DEGs. A total of 174 DEGs were identified, including 53 upregulated genes and 121 downregulated genes (Figure 2A). The top 10 up- and downregulated significant DEGs are listed in Supplementary Tables 2, 3.

The heat map and hierarchical clustering analysis demonstrated that samples in the EA groups were clustered together and separated from the MCAO model groups. These findings further suggest that EA treatment could modify gene expression changes in the MCAO model and that there were significant DEGs between the EA groups and the MCAO control group (Figure 2B).

### Gene Ontology Functional Enrichment Analysis of Differentially Expressed Genes

**Figure 3:**
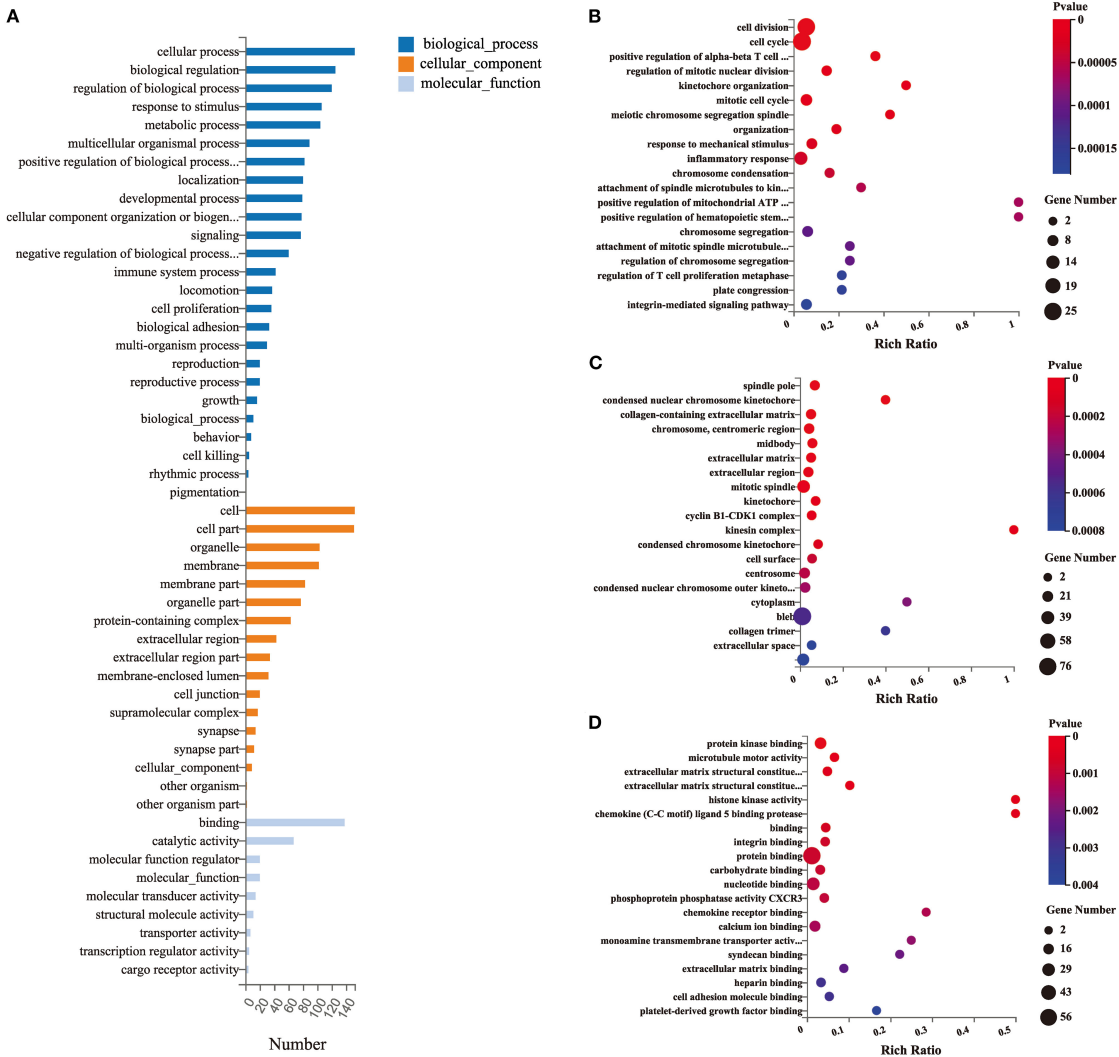
Gene ontology (GO) functional enrichment analysis of DEGs. **(A)** Classification of GO functional enrichment terms. **(B–D)** Bubble plots showing the top 20 significant biological processes, cellular components, and molecular functions of DEGs. Each bubble with varied color represents different *p*-value. *n* = 3 mice/group.

GO functional enrichment analysis (including BP, biological process; CC, cellular component; and MF, molecular function) was performed with 174 DEGs to predict the underlying biological function of DEGs between the EA and MCAO control groups. The DEGs were enriched to 50 subclasses of GOs. GO functional enrichment also revealed that the top 10 BPs were cellular process, biological regulation, regulation of biological process, response to stimulus, metabolic process, multicellular organismal process, positive regulation of biological process, localization, developmental process, and cellular component organization or biogenesis. The top 10 enriched CCs were cell, cell part, organelle, membrane, membrane part, organelle part, protein-containing complex, extracellular region, extracellular region part, and membrane-enclosed lumen. The top enriched MFs were binding, catalytic activity, molecular function regulator, molecular-function, molecular transducer activity, structural molecule activity, transporter activity, transcription regulator activity, and cargo receptor activity. The specific details are revealed in Figure 3.

### Pathway Enrichment Analysis of Differentially Expressed Genes

**Figure 4:**
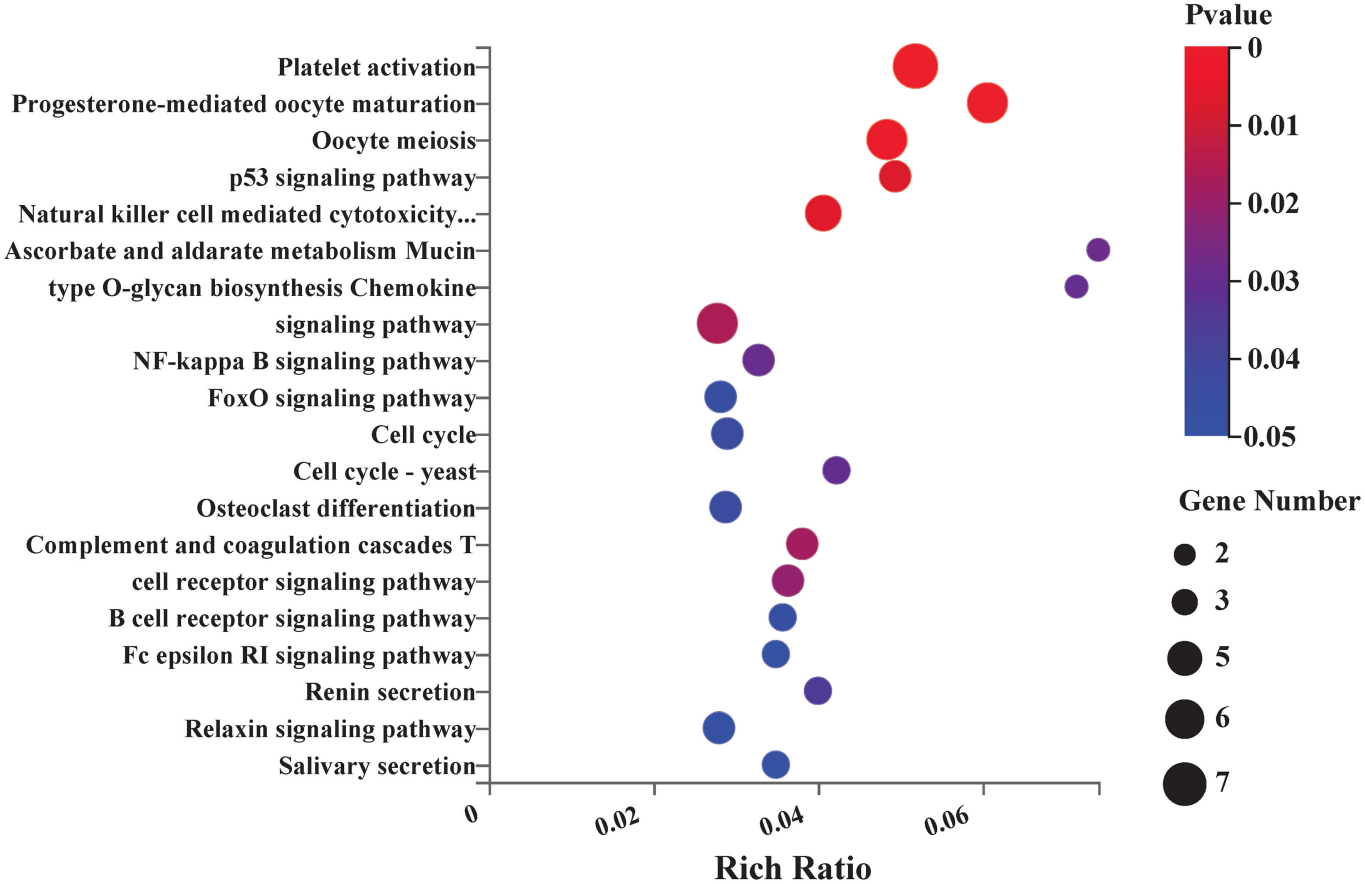
Kyoto Encyclopedia of Genes and Genomes (KEGG) pathway enrichment analysis between EA and control group. Bubble plots showing the top 20 significant pathways of DEGs. Larger bubbles illustrated higher number of genes. The color of each bubble represents the varied *p*-value. *n* = 3 mice/group.

KEGG pathway analysis was conducted to analyze 174 DEGs between the EA and model groups. The top KEGG pathways with the most enrichment were involved in platelet activation, p53 signaling pathway, natural killer cell-mediated cytotoxicity, cell cycle, FOXO signaling pathway, NF-kappa B signaling pathway, T-cell receptor signaling pathway, chemokine signaling pathway, complement, and coagulation cascades. The top 20 enrichment pathways are shown in Figure 4.

### Protein–Protein Interaction Network

**Figure 5:**
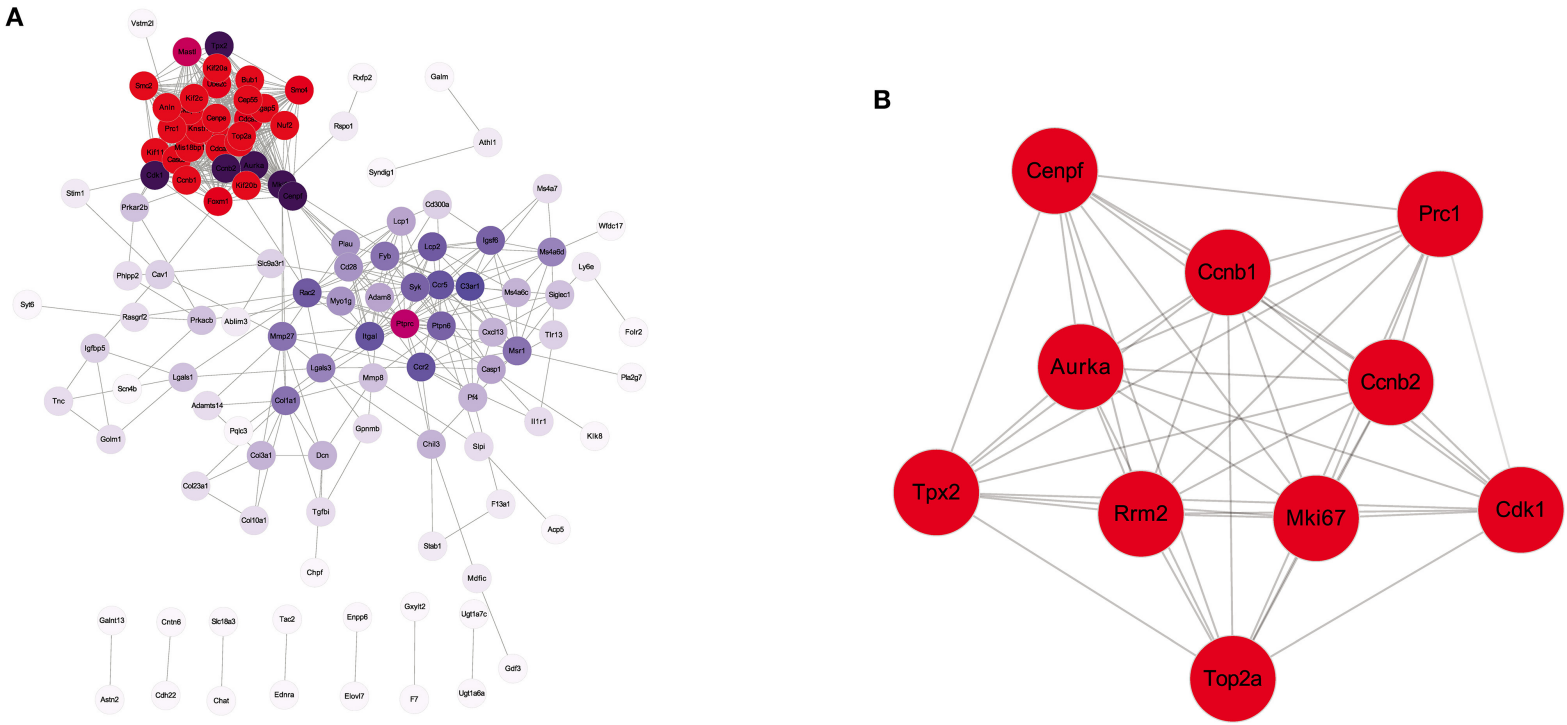
Protein–protein interaction (PPI) network analysis of the DEGs identified from EA + MCAO vs. MCAO group. **(A)** PPI network of all the DEGs. **(B)** The interaction network of top 10 hub genes. *n* = 3 mice/group.

To further visualize the interactions between the DEGs, a PPI network based on the STRING database was constructed. The network, comprising 118 nodes and 697 edges, was visualized by Cytoscape (Figure 5A). There were 27 genes with connectivity degrees larger than 30 based on the node degree method. The top 10 genes with the highest degree scores were regarded as hub genes, including Mki67, Cdk1, Tpx2, Cenpf, Ccnb2, Aurka, Prc1, Top2a, Ccnb1, and Rrm2. The specific information of the top 10 hub genes and corresponding degree scores are listed in Supplementary Table 4. In addition, the interactions of 10 hub genes were also reconstructed in Cytoscape (Figure 5B).

### Biological Analysis of Hub Gene Prediction and Validation

**Figure 6:**
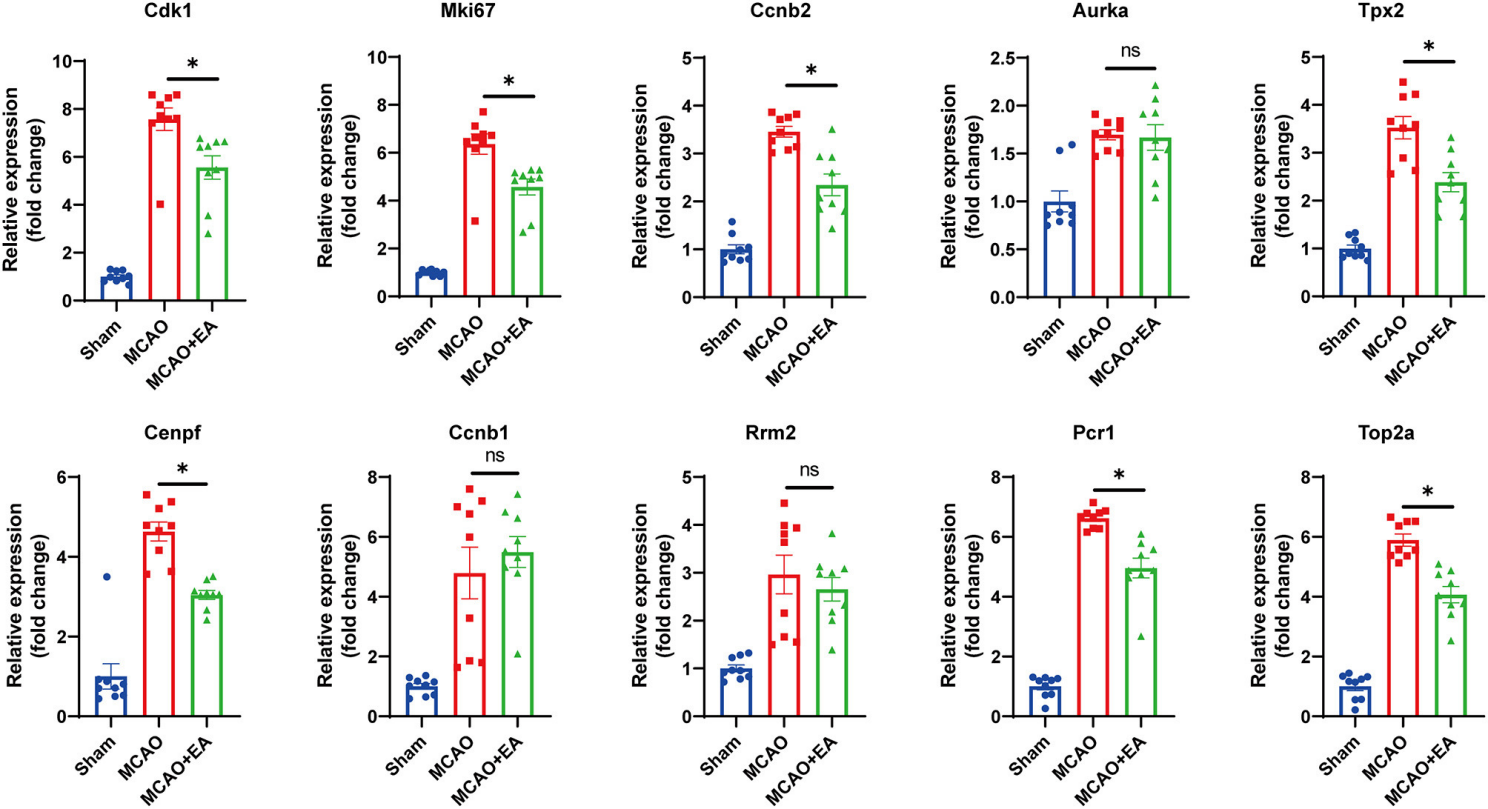
Validation of 10 hub genes with RT-qPCR. Statistical graph shows fold change of mRNA expression of DEGs regulated by EA treatment, normalized to β-actin level. Data are represented as means ± SEM, *n* = 3 mice/group (with three technical replicates); One-way ANOVA followed by Bonferroni (if equal variances assumed) or Dunnett's T3 (if equal variances not assumed) *post-hoc* test was used for statistical analysis, *p < 0.05, ^ns^*p* > 0.05.

Hub genes were verified by the RT-qPCR method. The Mki67, Cdk1, Tpx2, Cenpf, Ccnb2, Prc1, and Top2a genes were expressed at lower levels in the EA group than in the MCAO control group, which was consistent with the RNA-seq results (all p < 0.05). These results suggest that the RNA-seq results were reliable and reproducible (Figure 6).

### Prediction and Validation of the Upstream miRNAs of the Hub Genes

**Figure 7:**
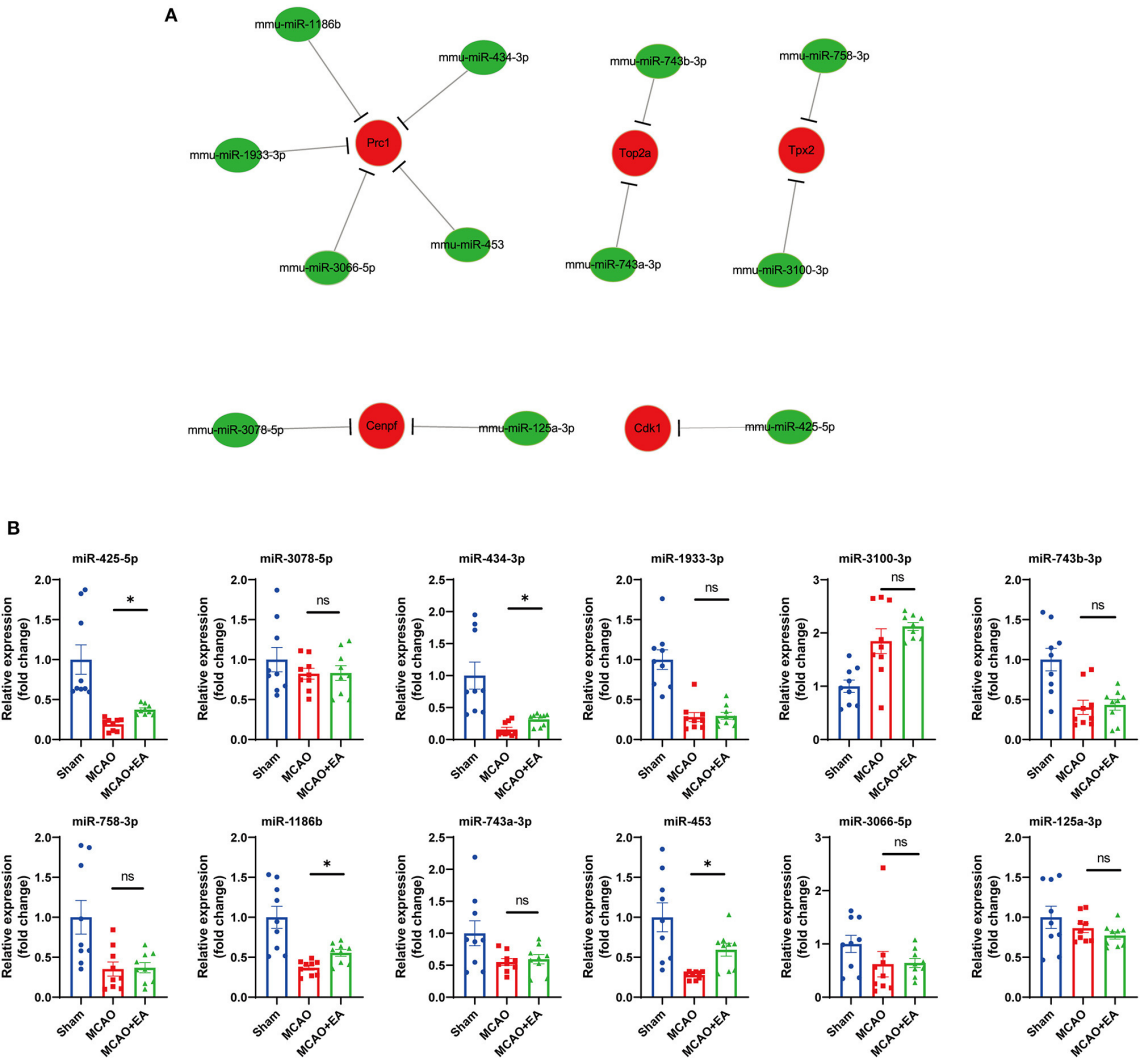
Prediction and validation of upstream miRNAs of hub genes. **(A)** Construction of miRNA-gene network using Cytoscape. **(B)** Validation of 12 predicted miRNA with RT-qPCR. Statistical graph shows fold change of miRNA expression regulated by EA treatment, normalized to U6 level. Data are represented as means ± SEM, *n* = 3 mice/group (with three technical replicates). One-way ANOVA followed by Bonferroni (if equal variances assumed) or Dunnett's T3 (if equal variances not assumed) *post-hoc* test was used for statistical analysis. *p < 0.05, ^ns^*p* > 0.05.

Next, we predicted the upstream miRNAs of the 10 hub genes by using the miRTarBase database. The results showed that a total of 12 miRNAs might regulate the expression of five target genes (Cdk1, Tpx2, Cenpf, Prc1, and Top2a) (see Figure 7A). Upstream miRNAs of the other five hub genes (Mki67, Ccnb2, Aurka, Ccnb1, and Rrm2) were not detected by miRTarBase. Moreover, we validated upstream miRNAs of target key genes using RT-qPCR, which showed that miR-425-5p, miR-453, miR-1186b, and miR-434-3p miRNAs were upregulated in the EA group (Figure 7B). Based on the classical inverse relationship between miRNAs and target genes, we further validated that miR-425-5p, miR-453, miR-1186b, and miR-434-3p miRNAs were regarded as key upstream miRNAs that negatively regulated key downstream genes.

### Construction of a Key miRNA–mRNA Network in Ischemic Stroke

**Figure 8:**
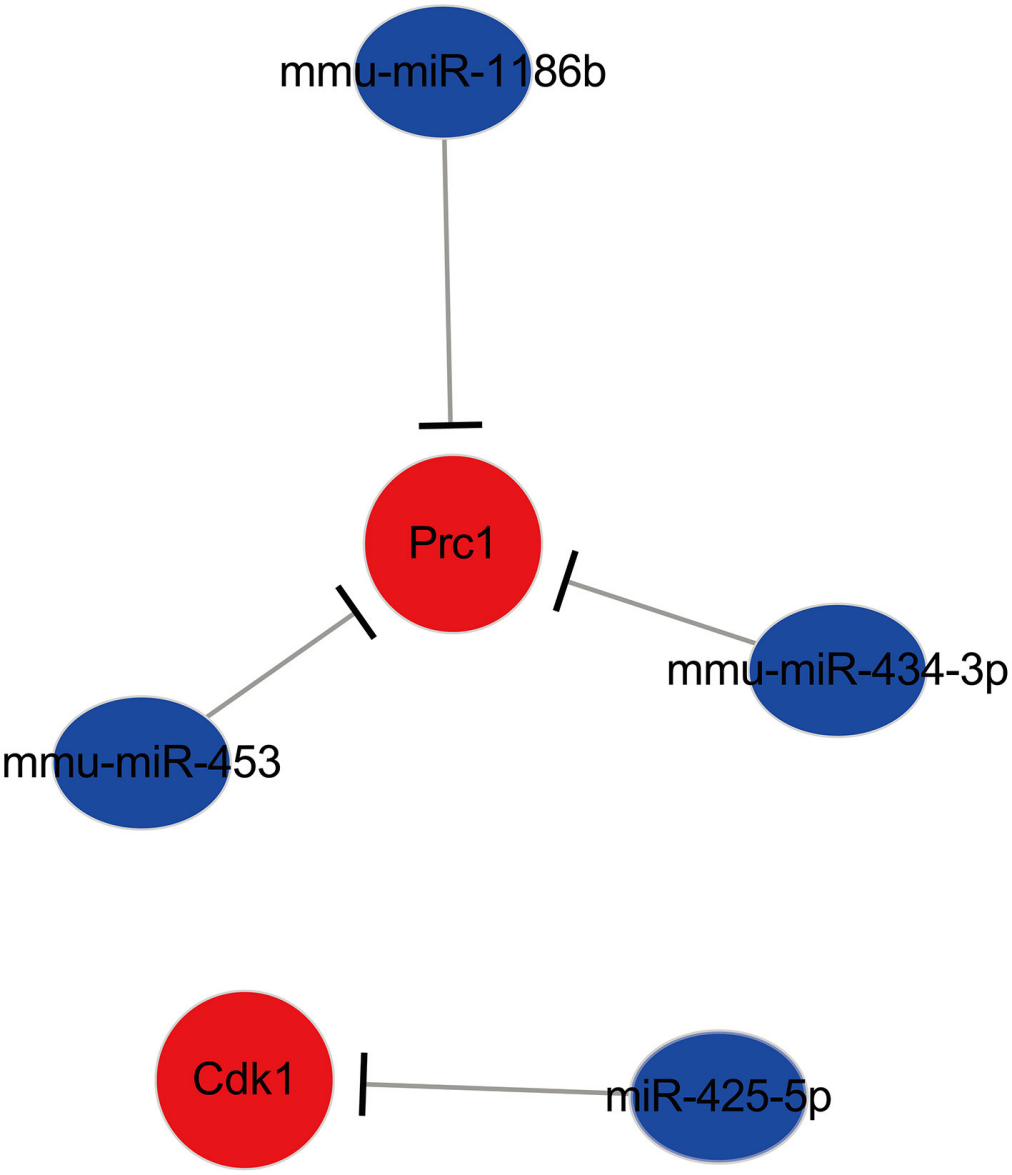
The key miRNA–mRNA regulated network in ischemic stroke modified by EA treatment.

After comprehensive analysis, a key miRNA–mRNA regulatory network in ischemic stroke modified by EA treatment was constructed. The network consisted of 12 miRNA–mRNA pairs. Among them, four miRNA–mRNA subnetworks were identified as key miRNA–mRNA networks, including miR-425-5p-Cdk1, mmu-miR-1186b-Prc1, mmu-miR-434-3p- Prc1, and mmu-miR-453-Prc1. The specific network is shown in Figure 8.

### The Localization of miRNA Network Regulated by Electroacupuncture in Ischemic Stroke

**Figure 9:**
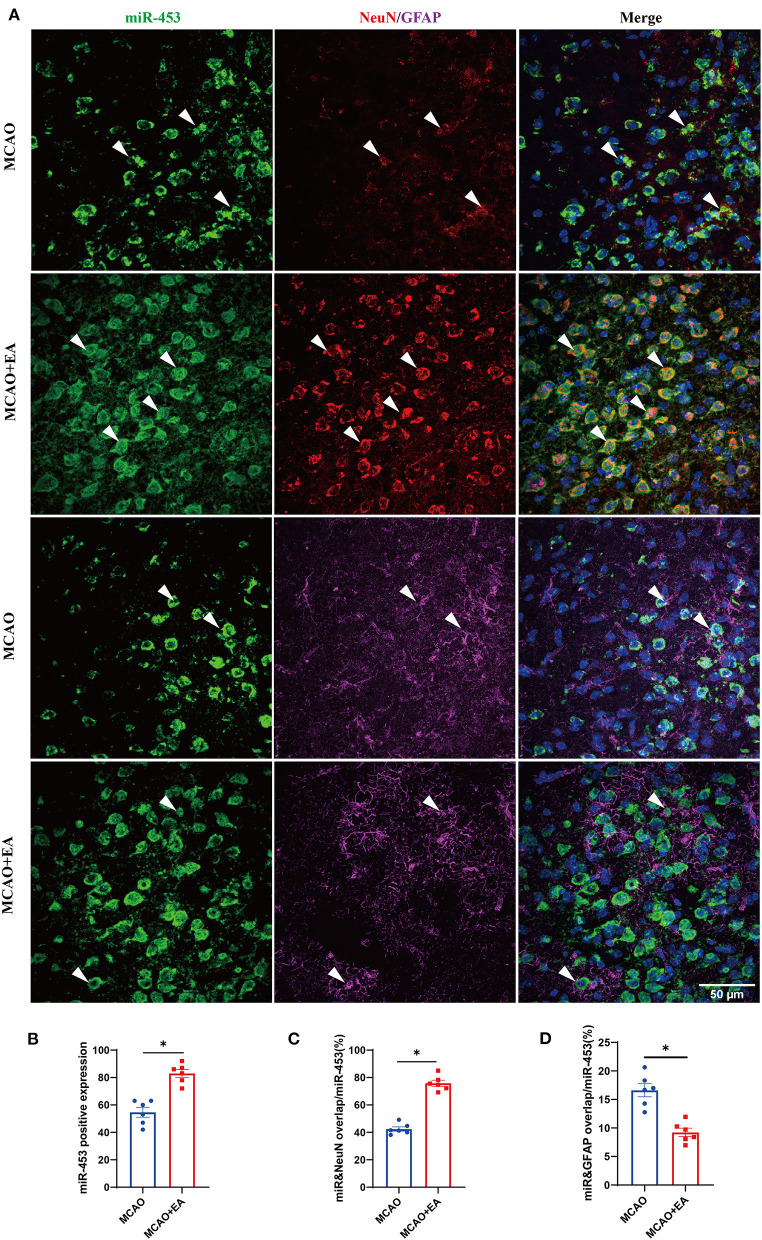
The localization of miRNA (miR-453) regulated by EA in ischemic stroke. **(A)** Confocal microscopic images showing the localization of miRNA (miR-453) (represented in green) with neuronal marker (NeuN, red) and astrocytic marker (GFAP, magenta) in the brain cortex regions of mice subjected to MCAO + EA intervention and MCAO control. The co-localization regions were indicated with arrows. Scale bar: 50 μm. **(B–D)** Quantification of miRNA (miR-453) immunoreactivity in neurons and astrocytes. **(B)** The expression of positive miRNA (miR-453). **(C)** The percentage of miRNA (miR-453) and NeuN co-localization/ miRNA (miR-453) expression. **(D)** The percentage of miRNA (miR-453) and GFAP co-localization/miRNA (miR-453) expression. Data are represented as means ± SEM, *n* = 6 regions/group; unpaired *t*-test was used. *p < 0.05, ^ns^*p* > 0.05.

The locations of miR-425-5p, miR-1186b, miR-434-3p, and miR-453 were evaluated in the brain by FISH with the neuronal marker (NeuN) and astrocytic marker (GFAP). The results revealed that the expression of miR-425-5p, miR-1186b, miR-434-3p, and miR-453 all significantly increased in the EA group compared with that in the MCAO control group (Figure 9B and Supplementary Figure 2B−4B). The results also showed that increased miR-425-5p, miR-1186b, miR-434-3p, and miR-453 were localized mainly in the NeuN-positive neurons of mice treated with EA intervention (Figure 9 and Supplementary Figures 2–4). In particular, the expressions of miR-425-5p, miR-1186b, miR-434-3p, and miR-453 were more abundant in the cortical neurons of mice in the EA group than those in the MCAO control group. Furthermore, miRNAs in both groups were localized mainly in the NeuN-positive neurons of mice (all larger than 45%), which indicated that these miRNAs might belong to neuronal-specific miRNAs. Instead, the expression of miR-425-5p, miR-1186b, miR-434-3p, and miR-453 in GFAP-positive cells decreased in the EA group compared with control group. However, all the miRNAs expressed in the GFAP-positive astrocytes of mice in both groups were lower than those in NeuN-positive neurons (all lower than 26%), suggesting that the expressions of miR-425-5p, miR-1186b, miR-434-3p, and miR-453 regulated by EA treatment were much more in neurons than in astrocytes (Figure 9 and Supplementary Figures 2–4).

## Discussion

From the behavioral test, we further verified that EA could enhance neurological function and sensorimotor recovery in ischemic stroke. EA could also decrease the percentage of infarct area and neuron loss induced by ischemic stroke. However, the potential complicated molecular mechanisms of EA in ischemic stroke remain unclear. Identification of key genes and targeted miRNAs and the construction of an miRNA–mRNA network regulated by EA intervention in ischemic stroke will facilitate the understanding of the EA treatment mechanisms and benefit the application of the present therapy.

In this study, we identified a total of 174 DEGs (53 genes upregulated and 121 genes downregulated) between the EA intervention and MCAO control groups by performing RNA-seq analysis. These results indicate that EA intervention could significantly modify gene expression changes in the MCAO model and thereby regulate the pathological and behavioral changes caused by ischemic stroke.

As shown by the GO and KEGG pathway analysis, the DEGs between the EA and model groups were enriched in cellular processes, regulation of biological processes, and response to stimulus in the BP category. They were enriched in extracellular region and membrane-enclosed lumen in the CC category, and they were enriched in binding-related term and molecular activity (transducer or transporter activity, transcription regulator activity, and cargo receptor activity) in the MF category. The KEGG pathway analysis indicated that the mechanism by which EA affects the MCAO model might mainly involve the regulation of related pathways, including the p53 signaling pathway, natural killer cell-mediated cytotoxicity, FOXO signaling pathway, and NF-kappa B signaling pathway. Previous studies also demonstrated that electroacupuncture could alleviate brain ischemia injury and contribute to neuroprotection against cerebral ischemia mainly by interacting with the NF-kappaB signaling pathway (35), PI3K/Akt/p53 pathway (36), and SIRT1-FOXO1 signaling pathway (37), which were in accordance with our RNA-seq analysis results. Furthermore, other pathways predicted by RNA-seq are still essential to explore the potential mechanism of EA therapy. For example, in the T-cell receptor signaling pathway, recent studies have reported that brain regulatory T cells are likely to provide a therapeutic strategy for neuronal protection against ischemic stroke (38, 39). These studies combined with our RNA-seq results help us to interpret that the T-cell receptor signaling pathway might play a critical role and might be a potential new target pathway for EA treatment of ischemic stroke.

To comprehensively explore the potential interaction and functions of significant DEGs regulated by EA in ischemic stroke, PPI networks were constructed, and hub genes were screened by the STRING database. The results showed the top 10 hub genes, and they were screened based on the degree method. Ultimately, seven downregulated hub genes (including Mki67, Cdk1, Tpx2, Cenpf, Ccnb2, Prc1, and Top2a) were identified as key genes regulated by EA treatment in ischemic stroke by PCR validation. Most of the key genes were closely related to ischemic stroke. Among these genes, Mki67, Top2a, and Cenpf, which are cellular markers for proliferation, were increased in the MCAO model and decreased by EA treatment. This was consistent with previous RNA-seq reports of ischemic stroke (40, 41). Previous studies also reported that following ischemic injury, the number of reactive astrocytes increased, and subsequently, microglia were activated since oxidative radical imbalance occurred (41). Overproliferation of these cell types promotes cytokine release, thus, leading to apoptosis of neurons and impeding neural regeneration (42–44). Therefore, EA might exert neuroprotection in ischemic stroke by inhibiting reactive astrocyte and microglial (Mki67, Top2a, and Cenpf as markers) proliferation. Cdk1 and Ccnb2 are associated with cell cycle signaling, and they are upregulated and promote neuronal cell death in OGD and MCAO models. This indicates that inhibition of cell cycle genes (Cdk1 and Ccnb2 as markers) could provide neuroprotection against ischemic neuronal death (45, 46). Prc1 is also involved in cell proliferation, causing an abnormal cell cycle, and the Prc1-mediated cascade pathway is also associated with neuronal necrosis in ischemic stroke (47, 48). A study of Tpx2 also indicated that inhibition of Tpx2 speeded the growth process of axons in cultured neurons, suggesting that the expression of Tpx2 likely regulates neuronal regeneration in ischemic stroke (49). These previous studies all further support that the mechanism of effect of EA in ischemic stroke may occur through downregulating these key genes.

MicroRNAs could negatively regulate downstream target gene expression by binding to the 3′UTRs of mRNA and contribute to the RNA-mediated regulatory network in ischemic stroke (50–52). Twelve upstream miRNAs that regulate key genes were predicted by the miRTarBase database. RT-qPCR validation revealed that mice in the EA group had high expression of four miRNAs (mmu-miR-425-5p, mmu-miR-1186b, mmu-miR-434-3p, and mmu-miR-453) that have a positive effect on ischemic stroke. The mmu-miR-425-5p has been shown to attenuate apoptosis and suppress necroptosis, oxidative stress, and inflammation by restraining the PIPK1 signaling axis (53, 54). The miR-425-5p could possibly be useful in controlling the negative effects of ischemic stroke, as Lim reported (55). The mmu-miR-434-3p has been suggested to potentially increase neuronal regeneration under conditions of stress, especially in brain injury (56–58). The miR-453 was found to be a good outcome associated with neural repair during ischemic stroke recovery (59, 60). Therefore, combining these previous studies and our validations, we infer that these four miRNAs might be regarded as recovery biomarkers regulated by EA in ischemic stroke. Furthermore, FISH and IF revealed that these increased miRNAs regulated by EA mainly occur in neuronal cells of cortex and less in GFAP-positive cells, demonstrating that EA might exert a potential protective effect of ischemic stroke through regulating these miRNAs in brain neuronal cells.

Overall, key miRNA-mRNA networks were identified based on RNA-seq and RT-qPCR validations, and we found that miR-425-5p-Cdk1, mmu-miR-1186b-Prc1, mmu-miR-434-3p- Prc1, and mmu-miR-453-Prc1 were crucial networks regulated by EA in brain neuronal cells of ischemic stroke. The mechanism by which EA exerts a neuroprotective effect in ischemic stroke might involve regulating these key miRNA–mRNA networks (by upregulating these upstream miRNAs and thereby targeting downstream genes). However, because of the complicated molecular mechanisms of EA in ischemic stroke, more animal experiments investigating the miRNA–mRNA networks regulated by EA are still needed for further verification.

## Conclusion

In summary, bioinformatics analysis and experimental verification were performed. We identified seven key genes and four miRNAs that possessed significant values for EA treatment in ischemic stroke. In addition, four novel miRNA–mRNA networks were constructed as potential molecular mechanisms underlying the therapeutic effects of EA on ischemic stroke. These findings provide some key clues for further understanding and investigating the underlying mechanisms of EA treatment for ischemic stroke in the future. However, more experimental validations related to miRNA–mRNA networks are still needed to further confirm these findings.

## Data Availability Statement

RNA sequencing data has been deposited in the NCBI Trace Archive NCBI Sequence Read Archive (SRA) repository. The accession number is BioProject ID: PRJNA760242.

## Ethics Statement

The animal study was reviewed and approved by Animal Welfare and Ethics Committee of Guangzhou University of Chinese Medicine.

## Author Contributions

MZ, DC, and HL designed the study. XL, YX, HG, and ZH acquired the data. CW, LZ, and QW analyzed and interpreted the data. CW drafted the manuscript. MZ and DC revised the manuscript for important intellectual content. All authors read and approved the final manuscript.

## Funding

This study was supported by the China Postdoctoral Science Foundation (No. 2020M682684), National Natural Science Foundation of China (No. 82104978), Natural Science Foundation of Shenzhen (No. JCYJ20190807112405520), Bao'an TCM Development Foundation (No. 2020KJCX-KTYJ-130), Bao'an TCM Development Foundation (No. 2020KJCX-KTYJ-134), and Natural Science Foundation of Guangxi Province (No. 2018GXNSFAA294030).

## Conflict of Interest

The authors declare that the research was conducted in the absence of any commercial or financial relationships that could be construed as a potential conflict of interest.

## Publisher's Note

All claims expressed in this article are solely those of the authors and do not necessarily represent those of their affiliated organizations, or those of the publisher, the editors and the reviewers. Any product that may be evaluated in this article, or claim that may be made by its manufacturer, is not guaranteed or endorsed by the publisher.
