# Supplementary material for: Integrated Bioinformatics Analysis of Potential mRNA and miRNA Regulatory Networks in Mice With Ischemic Stroke Treated by Electroacupuncture
Source: Front Neurol. 2021 Sep 10;12:719354. doi: 10.3389/fneur.2021.719354 (PMC8461332; doi:10.3389/fneur.2021.719354)
Supplement: Supplementary file 1 [file Data_Sheet_1.DOCX]

**Supplementary file Online Content**

[**Supplementary file Table S1**: Detailed primer sequences of genes and miRNA 2](#_Toc77707431)

[**Supplementary file Table S2:** Detailed information of the top 10 upregulated mRNAs in MCAO brain regulated by EA 3](#_Toc77707432)

[**Supplementary file Table S3**: Detailed information of the top 10 downregulated mRNAs in MCAO brain regulated by EA 4](#_Toc77707433)

[**Supplementary file Table S4**: Detailed information of the top 10 hub genes and corresponding degree score in MCAO brain regulated by EA 4](#_Toc77707434)

[**Supplementary file Figure S1**. Quantitative analysis of gene expression. 5](#_Toc77707435)

[**Supplementary file Figure S2**. The localization of miR-434-3p regulated by EA in ischemic stroke 6](#_Toc77707436)

[**Supplementary file Figure S3**. The localization of miR-425-5p regulated by EA in ischemic stroke 8](#_Toc77707437)

[**Supplementary file Figure S4**. The localization of miR-1186b regulated by EA in ischemic stroke 10](#_Toc77707438)

# Supplementary file Table S1: Detailed primer sequences of genes and miRNA

Table1a. Detailed primer sequences of genes

| Gene | Sequences |
| --- | --- |
| Mki67 | FORWARD: CACAGAGAACAAAGGTGTGAAG  REVERSE: GGAGACTGCAGAGCTATTTTTG |
| Cdk1 | FORWARD: AGTTCATGGATTCTTCACTCGT  REVERSE: GTGTGTACACTCGTATCGGTAT |
| TPX2 | FORWARD: TGATGAAGCAGCTTCTACGTAT  REVERSE: CACAGCCCTTGTACGATATTTG |
| Cenpf | FORWARD: TGATGAAGCAGCTTCTACGTAT  REVERSE: CACAGCCCTTGTACGATATTTG |
| Ccnb2 | FORWARD: ATTACACAGGCTACATGGAGAG  REVERSE: GGTACGGTTGTCATTGACTTTC |
| Aurka | FORWARD: GCAGTGGACTTTGGAAGATTTT  REVERSE: CAGGATGAACTTGCTTTGTCTC |
| Prc1 | FORWARD: CTGTATGCCGCCCTGTGATGTG  REVERSE: CACGCCTCGATTCCTTTGTCTCC |
| Top2a | FORWARD: TATGACTCAGCACAAGCAGCAAGG  REVERSE: GGATCTCGTGTTGGGAAGGCATC |
| Ccnb1 | FORWARD: GCTCTTCCAGGGGTGTGCTTTG  REVERSE: GGTGGCATTACAAGACAGGAGTGG |
| Rrm2 | FORWARD: CCGGTTCTTTTGCATCGATATT  REVERSE: GGAACTCCTGCTCTATCCTAAC |
| β-actin | FORWARD: CTACCTCATGAAGATCCTGACC  REVERSE: CACAGCTTCTCTTTGATGTCAC |

Table1b. Detailed primer sequences of genes and miRNA

| miRNA | Sequences |
| --- | --- |
| mmu-miR-425-5p： | CAATGACACGATCACTCCCGTTGA |
| mmu-miR-3100-3p | CTGTGACACACCCGCTCC |
| mmu-miR-758-3p | CGCTTTGTGACCTGGTCCACTA |
| mmu-miR-3078-5p | TCAAAGCCTAGACTGCAGCTACCT |
| mmu-miR-125a-3p | ACAGGTGAGGTTCTTGGGAGC |
| mmu-miR-3066-5p | CGCGCTTGGTTGCTGTAGATTAAGTAG |
| mmu-miR-453 | AGGTTGCCTCATAGTGAGCTTGCA |
| mmu-miR-434-3p | CGTTTGAACCATCACTCGACTCCT |
| mmu-miR-1933-3p | TGCCAGGACCATCAGTGTGACTAT |
| mmu-miR-743a-3p | GCGAAAGACACCAAGCTGAGTAGA |
| mmu-miR-743b-3p | GCGCGAAAGACATCATGCTGAATAGA |
| mmu-miR-1186b | CCTGGGATTAAAGGCATGCACCA |

# Supplementary file Table S2: Detailed information of the top 10 upregulated mRNAs in MCAO brain regulated by EA

| Gene ID | Gene Symbol | log2 (EA / Model) | Qvalue (EA / Model) |
| --- | --- | --- | --- |
| 12647 | Chat | 3.498087108 | 0.049711023 |
| 20508 | Slc18a3 | 2.958238498 | 0.029662471 |
| 100384868 | Gm37013 | 1.660513714 | 0.039232343 |
| 140498 | Rxfp2 | 1.652803724 | 0.036319884 |
| 240058 | Cpne5 | 1.625104945 | 1.36E-04 |
| 245038 | Dclk3 | 1.589873678 | 0.007155226 |
| 54524 | Syt6 | 1.448431713 | 0.004155462 |
| 399548 | Scn4b | 1.223208616 | 0.008365023 |
| 75141 | Rasd2 | 1.213305254 | 0.037152442 |
| 53417 | Hif3a | 1.199806147 | 0.004155462 |

# Supplementary file Table S3: Detailed information of the top 10 downregulated mRNAs in MCAO brain regulated by EA

| Gene ID | Gene Symbol | log2 (EA / Model) | Qvalue (EA / Model) |
| --- | --- | --- | --- |
| 26941 | Slc9a3r1 | -0.431382708 | 0.034855368 |
| 17069 | Ly6e | -0.436957988 | 0.002221129 |
| 74551 | Pck2 | -0.439278015 | 0.032386024 |
| 109246 | Tspan9 | -0.526587515 | 0.002221129 |
| 27226 | Pla2g7 | -0.534544947 | 5.00E-04 |
| 16011 | Igfbp5 | -0.621669627 | 0.031056025 |
| 14211 | Smc2 | -0.719511697 | 0.037152442 |
| 74559 | Elovl7 | -0.801272772 | 0.002236122 |
| 212167 | Gsap | -0.839295514 | 0.027648272 |
| 212974 | Pgghg | -0.85344513 | 0.036319884 |

# Supplementary file Table S4: Detailed information of the top 10 hub genes and corresponding degree score in MCAO brain regulated by EA

| Rank | Symbol | Degree score |
| --- | --- | --- |
| 1 | Mki67 | 38 |
| 2 | Cdk1 | 34 |
| 3 | Tpx2 | 32 |
| 4 | Cenpf | 32 |
| 5 | Ccnb2 | 32 |
| 6 | Aurka | 32 |
| 7 | Prc1 | 31 |
| 8 | Top2a | 31 |
| 9 | Ccnb1 | 31 |
| 10 | Rrm2 | 31 |

# Supplementary file Figure S1. Quantitative analysis of gene expression.


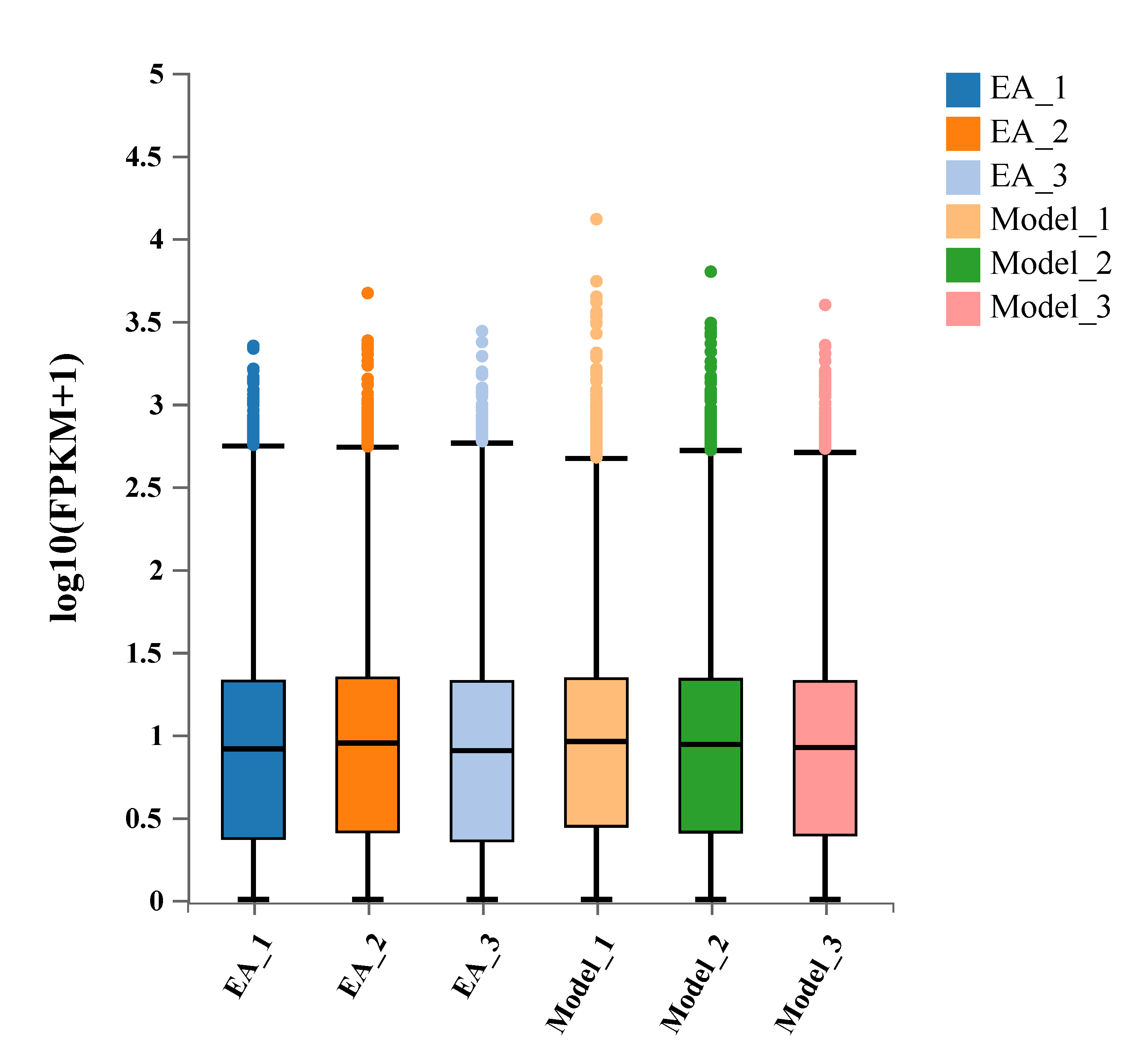


**Figure S1.** Quantitative analysis of gene expression**.** Boxplot shows the overall range and distribution of FPKM value of gene expression of all the samples.

# Supplementary file Figure S2. The localization of miR-434-3p regulated by EA in ischemic stroke


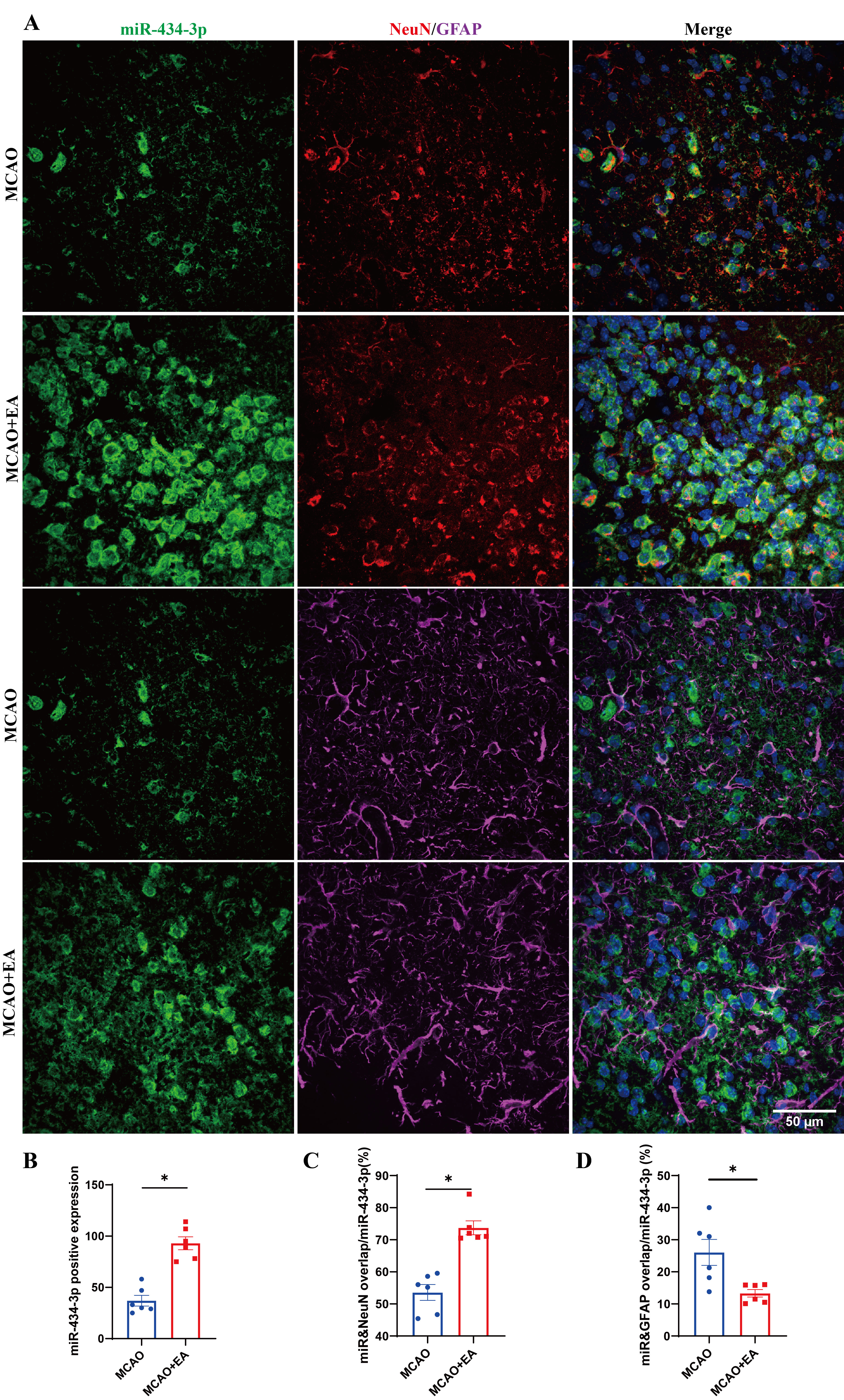


Figure S2. The localization of miR-434-3p regulated by EA in ischemic stroke. (A) Confocal microscopic images showing the localization of miRNA (represented in green) with neuronal marker (NeuN, red) and astrocytic marker (GFAP, magenta) in the brain cortex regions of mice subjected to MCAO+EA intervention and MCAO control. The co-localization regions were indicated with arrows. Scale bar: 50 µm. (B-D) Quantification of miR-434-3p immunoreactivity in neurons and astrocytes. (B) The expression of positive miR-434-3p. (C) The percentage of miR-434-3p & NeuN co-localization/ miR-434-3p expression. (D) The percentage of miR-434-3p & GFAP co-localization/ miR-434-3p expression. Data are represented as means±SEM, n=6 regions/group; Unpaired t test was used. **p*<0.05, ^ns^*p*>0.05.

# Supplementary file Figure S3. The localization of miR-425-5p regulated by EA in ischemic stroke


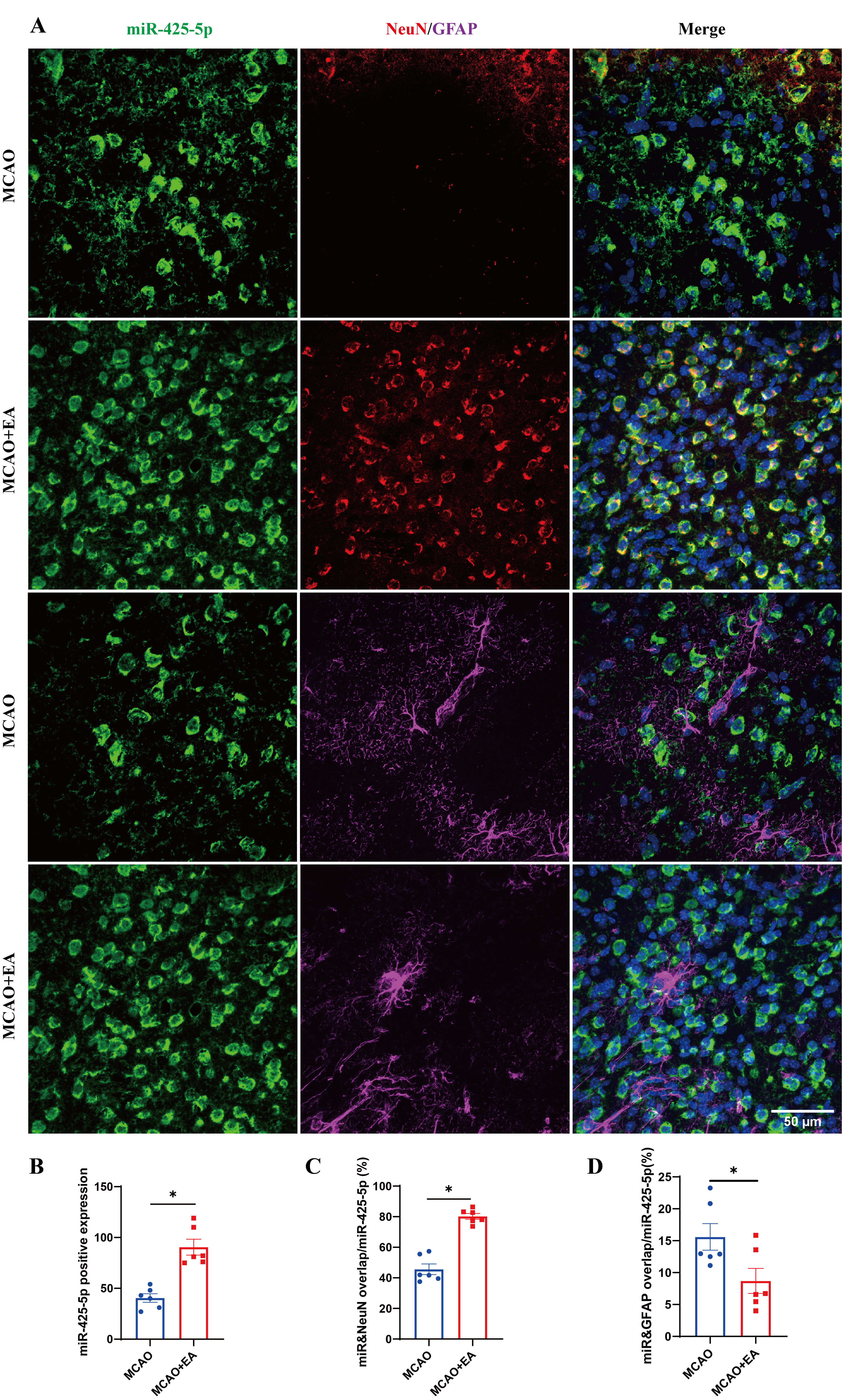


Figure S3. The localization of miR-425-5p regulated by EA in ischemic stroke. (A) Confocal microscopic images showing the localization of miRNA (represented in green) with neuronal marker (NeuN, red) and astrocytic marker (GFAP, magenta) in the brain cortex regions of mice subjected to MCAO+EA intervention and MCAO control. The co-localization regions were indicated with arrows. Scale bar: 50 µm. (B-D) Quantification of miR-425-5p immunoreactivity in neurons and astrocytes. (B) The expression of positive miR-434-3p. (C) The percentage of miR-425-5p & NeuN co-localization/ miR-425-5p expression. (D) The percentage of miR-425-5p & GFAP co-localization/ miR-425-5p expression. Data are represented as means±SEM, n=6 regions/group; Unpaired t test was used. **p*<0.05, ^ns^*p*>0.05.

# Supplementary file Figure S4. The localization of miR-1186b regulated by EA in ischemic stroke


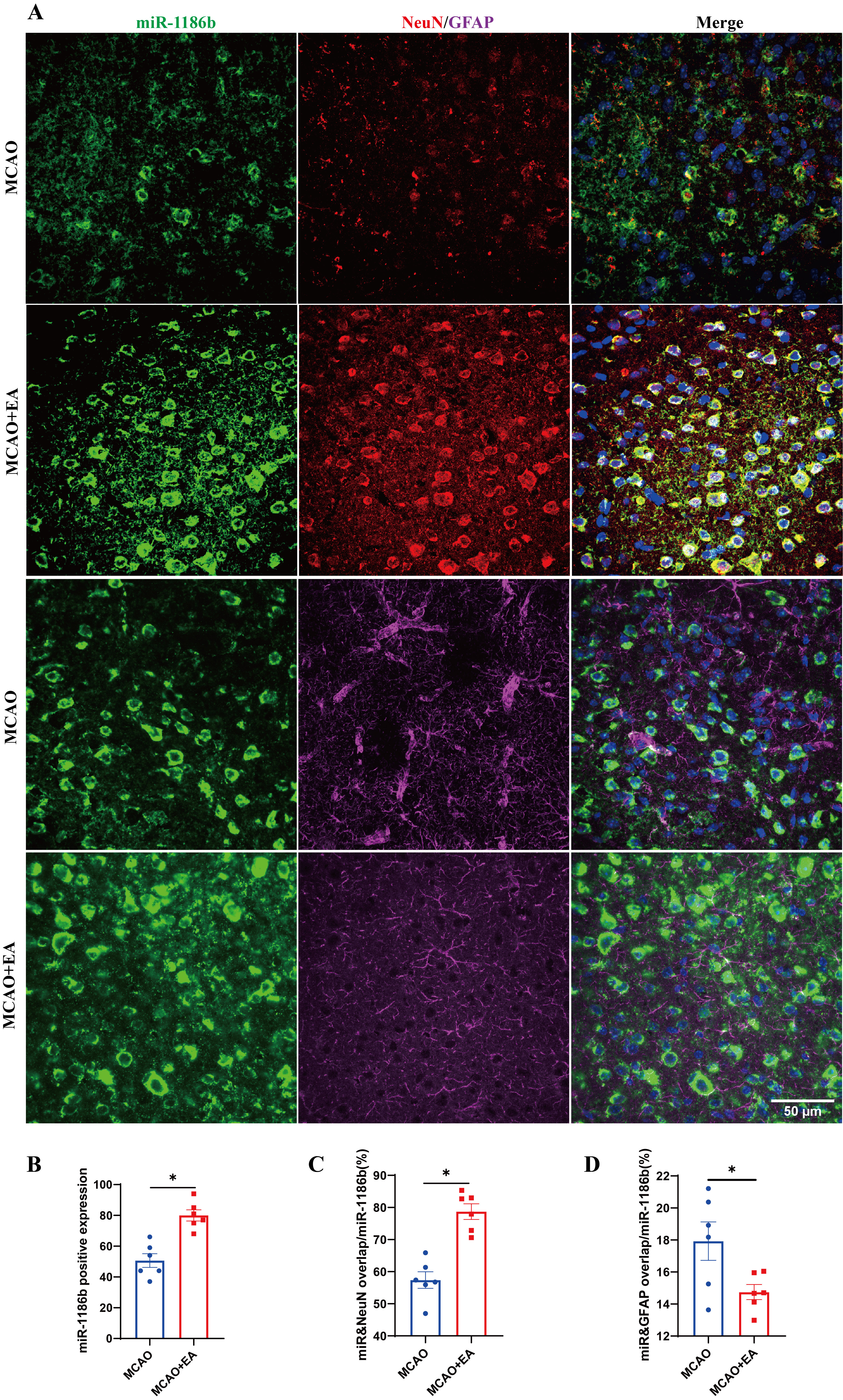


Figure S4. The localization of miR-1186b regulated by EA in ischemic stroke. (A) Confocal microscopic images showing the localization of miRNA (represented in green) with neuronal marker (NeuN, red) and astrocytic marker (GFAP, magenta) in the brain cortex regions of mice subjected to MCAO+EA intervention and MCAO control. The co-localization regions were indicated with arrows. Scale bar: 50 µm. (B-D) Quantification of miR-1186b immunoreactivity in neurons and astrocytes. (B) The expression of positive miR-1186b. (C) The percentage of miR-1186b & NeuN co-localization/ miR-1186b expression. (D) The percentage of miR-1186b & GFAP co-localization/ miR-1186b expression. Data are represented as means±SEM, n=6 regions/group; Unpaired t test was used. **p*<0.05, ^ns^*p*>0.05.
